# Interdisciplinary Learning in Mathematics and Science: Transfer of Learning for 21st Century Problem Solving at University

**DOI:** 10.3390/jintelligence8030032

**Published:** 2020-09-01

**Authors:** Yoshitaka Nakakoji, Rachel Wilson

**Affiliations:** Sydney School of Education and Social Work, University of Sydney, Sydney, NSW 2006, Australia; yoshitaka.nakakoji@sydney.edu.au

**Keywords:** 21 century skills, transfer, problem solving, mathematics, science, interdisciplinary, university, think-aloud

## Abstract

Transfer of learning, the application of learning to different contexts over time, is important to all learning for development. As 21st century skills specifically aim to be “generic,” there is an assumption that they can be transferred from context to context. We investigate the process of transfer in problem solving, with specific focus on mathematical problem solving tasks. Problem solving is highly valued in 21st century workplaces, where mathematical skills are also considered to be foundational in STEM and of paramount importance. This study examines the transfer of first semester mathematics learning to problem solving in second semester physics at university. We report on: (1) university students’ (n = 10) “think-aloud” accounts of the process of transfer; and (2) students’ (n = 10) and academics’ (n = 8) perspectives on transfer processes and problem solving. Think-aloud accounts show students’ recursive use of interpretation, integration, planning and execution thinking processes and highlight the meta-cognitive strategies used in transfer. Academics’ and students’ perspectives on transfer show disparities. Understanding these perspectives is important to current initiatives to integrate and optimise 21st century learning within universities. We argue that renewed attention on the concept of transfer is needed if the generic aims of 21st century skills are to be understood and promoted.

## 1. Introduction

This article focuses on the centrality and potential of transfer of learning for 21st Century (21C). In particular we explore mathematics transfer, which is widely recognised as central to human development, educational systems and economies, and has recently been the focus of many research projects, policy and public campaigns internationally (Australian Industry Group 2013; Office of the Chief Scientist 2013; National Research Council 2013; The Royal Society Science Policy Centre 2014; U.S. Congress Joint Economic Committee 2012; U.S. Department of Education 2016). However, given its importance, relatively little recent research exists that examines how mathematics learning is applied in other contexts; and even less research that operationalises and explores transfer of learning within authentic educational systems (Nakakoji and Wilson 2018). We argue that considering the transfer of mathematics learning is critical to the development of 21C skills; and that attention to the role of transfer more broadly, within holistic conceptions of 21C learning, is needed to progress understanding in the field.

While looking at transfer between mathematics and physics, we also consider the relationship between mathematics and science more broadly within the context of university. The importance of this relationship to industry and society is undisputed; and learning in these disciplinary areas is seen as key to 21Century education. Yet, within universities, optimal productivity at the nexus between mathematics and science is often assumed and rarely examined. Most universities, for example, offer mathematics “service courses” to a wide range of degree programs and assume that learning in mathematics courses is effectively applied across degree curriculum. However, sparse research is published to support this assumption and less still that can inform practice in interdisciplinary teaching and learning (Nakakoji and Wilson 2018).

This paper makes two contributions. First, we examine students’ think-aloud accounts of the processes they use in transfer of learning tasks. Second, we examine both student and academic staff perspectives on the mathematics/science relationship, including their views on factors that promote or hinder transfer.

We take a process approach to examine transfer of learning, in relation to 21C problem solving skills. The process-oriented approach (Sternberg 2000) aims to “isolate a finite set of competent that can be combined in various ways to perform any cognitive task” (Meichenbaum 1980, p. 271). We adopt a think-aloud protocol to collect data and document the cognitive processes used in transfer of mathematics learning to science problem-solving tasks. This approach, in tandem with post-task interviews, has strong practical relevance to education because it can identify barriers and stumbling blocks to student learning.

This small-scale exploratory research is nonetheless significant given the historical relationship between mathematics and science and the relative lack of research attention given to this important interdisciplinary relationship. The study makes a preliminary contribution to applied understanding of what can be done to promote the transfer of learning for 21C skills at this important disciplinary nexus.

### 1.1. Transfer and 21st Century Learning

Rapid technological advancement has changed the skills and knowledge used in workplaces. The change requires employees to process various types of information, analyse big data, interact with and communicate with people, and apply their prior knowledge and experience to a new situation to solve the complex problem in different contexts. Industries demand graduates with generic skills, such as higher-order critical thinking and problem solving, and also strong metacognitive and communication skills. According to the Organization for Economic Co-operation and Development (OECD), 21st century skills refer to “skills and competencies young people will be required to have in order to be effective workers and citizens in the knowledge society of the 21st century” (Ananiadou and Claro 2009, p. 8). Problem solving is consistently identified as central to these requirements, and routinely listed as a desirable graduate attribute for employability and as an integral component in 21C learning.

Transfer is viewed as critical for future education and central to the application of 21st century skills (OECD 2018). Logic dictates that transfer is needed for the application of these generic and “transferable” skills—although this has not, as yet, been as widely acknowledged in the academic literature as might be expected. With predictions of rapidly evolving environments in our future, the ability to apply prior learning to new contexts is essential. Transfer of mathematical learning is the ability of students to apply mathematical skills, knowledge, and reasoning to other disciplines, and this is likely to be particularly important to 21st century skills. Demonstration of this ability is a central issue in mathematics and science education (Tariq 2013; King et al. 2015).

### 1.2. The Relationship between Mathematics and Science

National reports suggest many countries are concerned about participation, standards, and capacity building in mathematics and science for STEM related industry and labor markets (Australian Industry Group 2013; National Research Council 2013; Office of the Chief Scientist 2013; The Royal Society 2014; U.S. Department of Education 2016). This has increased research focus on education in these fields. However, learning in these fields is not discrete, they are intertwined, and we need to know more about how learning in mathematics and science is transferred and shared. The study presented here is part of a larger project exploring learning at the mathematics/science nexus at one Australian university (Nakakoji and Wilson 2014, 2018; Nakakoji et al. 2014).

Interdisciplinary relationships between mathematics and science are critically important as mathematics is applied in diverse disciplines and these relationships lead to advancements across disciplines (National Research Council 2013). Both mathematics and science educators need to consider the implications of this interdisciplinarity in terms of effective teaching and learning in schools and universities. For example, U.S. primary and secondary education standards require teachers to enhance the synergy between these disciplines by application of mathematics to science, e.g., mathematical modelling and statistics (Stage et al. 2013). The close relationship is also evident in the fact that mathematical learning in high schools and university is a strong predictor of attainment in science; with mathematics scores explaining between 43 and 87 percent of the variance in a range of science subjects (Nakakoji and Wilson 2014; Sadler and Tai 2007). Furthermore, there is currently a flux of research into STEM and internationally many universities conduct educational research in these disciplines in order to improve their teaching (King and Sen 2013). As part of this, it is widely recognised that effective communication and collaboration between academics in STEM disciplines is important (Anderson et al. 2011; Blumberg et al. 2005; Orton and Roper 2000).

The strong correlations found between mathematics and science learning (Nakakoji and Wilson 2014; Sadler and Tai 2007) can be attributed to both the underlying shared general and specific intelligence factors (Nisbett et al. 2012) and the transfer of specific learning and skills between these disciplines (Roberts et al. 2007; Nakakoji and Wilson 2018), including, for example, the common and shared use of problem solving schemata and other cognitive strategies. The g factor or general ability is the underlying foundation to diverse cognitive abilities that directly or indirectly affects all learning, including in mathematics and science. According to Cattell-Horn-Carroll theory, g is the strongest factor analytical construct in the hierarchical model of intelligence (see for example, Taub et al. 2008). Furthermore, the g factor has been shown to be highly correlated with international assessments of educational attainment, such as PISA and TIMSS, and IQ tests (Rindermann 2007). When examining two different educational attainments it is unsurprising to see high levels of correlation, due the fact that both will draw on the g factor.

The relationship between mathematics and general ability has been examined empirically and is particularly strong. The g factor was correlated with 25 secondary school subjects in the UK; and mathematics had the strongest association with g (r = 0.77), explaining approximately sixty percent of the variance in general ability (Deary et al. 2007). This suggests that among educational attainments, mathematics is particularly linked to g and that this might also explain how mathematics would be a strong predictor of other educational attainments.

Complicating this picture of correlated educational attainments is the unique relationship between mathematics and science. As these are highly cognate disciplines, they may share additional factors. These may contribute to g, and they may also be specific intelligence factors which explain additional achievement variance beyond *g*. Taub and colleagues (2008) have demonstrated how cognitive ability factors, including fluid reasoning and processing speed, are highly associated with mathematical attainment. Science and mathematics problem-solving assessments may share requirements for fluid reasoning and processing speed (which contribute to g). Science and mathematics may also both draw on specific factors, like gq (quantitative knowledge), so we might expect higher levels of correlation between them than between other, less cognate disciplines. We might also expect these factors to be drawn on in transfer of learning tasks (Richmond et al. 2011) and in problem solving tasks (Decker and Roberts 2015).

### 1.3. University Mathematics Service Courses and Science Courses

In many countries, including Australia, universities and higher education institutions adopt a “service course” model. In this model, first year mathematics courses are provided by mathematics departments to students from diverse STEM disciplines, such as biology, chemistry, engineering, and physics. These courses cover calculus, differential equations, and linear algebra; and can be offered at different levels (fundamental, intermediate, advanced) according to these students’ prior learning in high school mathematics and the requirements for their degree programs (see for example Nakakoji et al. 2014). As sciences are viewed as mathematically cognate disciplines, many institutions have mandatory requirements for science students to study first year mathematics service courses where mathematical skills, knowledge, and reasoning are developed so that they can be applied in other disciplinary learning. This approach often goes unchallenged, and yet it is built upon a range of assumptions, including: (i) students engage with service courses and effective learning occurs; (ii) skills and understanding in mathematics service courses are transferred to other disciplines; (iii) transfer of skills and understanding is assessed in other disciplinary areas, and (iv) the mathematics learnt in service courses is useful in building 21C skills for professions and working life. Interrogating these assumptions within one Australian university, we examined the correlation between mathematics and science learning (Nakakoji and Wilson 2014; Nakakoji et al. 2014) and looked for evidence of transfer of learning between them. Using extant university exam assessments, we were able to demonstrate transfer of learning in some science courses, but not others, dependent on the requirements for mathematical reasoning and calculation evident within the course assessments (Nakakoji and Wilson 2018). Specifically, we found assessment of mathematical learning and transfer of learning was evident in only engineering and physics course assessments; and mathematics was not assessed in biology and biochemistry courses in a way that enabled testing and demonstration of transfer from the mathematics service courses.

The larger project employed mixed method design to explore transfer of mathematical learning in a range of different ways (see Nakakoji et al. 2014), and findings demonstrated the importance and predictive power of mathematics to the subsequent learning in university science (Nakakoji and Wilson 2014, 2018). In Nakakoji and Wilson (2014), multiple regression analysis confirmed strong relationships between mathematics and science attainment. For example, 84% of the variance in second semester biology was explained by first semester biology and mathematics marks; with mathematics uniquely explaining 3.8 percent. In addition, in Nakakoji and Wilson (2018), transfer was quantitatively measured, using a previously established Transfer Index (Roberts et al. 2007). Our research applied this index to pre-existing university tests and exam data; demonstrating, for the first time, that these can be used to assess transfer. We were able to identify transfer of mathematics learning to physics and engineering; however, in biology and biochemistry, the papers provided no opportunity to assess transfer. Path analyses showed significant direct-transfer effect in the advanced physics course, i.e., the final marks in the course increased by a 0.67 standard deviation when transfer was increased by one standard deviation. This analysis relied on data from marks in exams and demonstrated the importance of mathematics to science learning; however, it did not examine the processes of transfer.

In this paper, we report on students’ think-aloud accounts of transfer and the views of students and academics from across this range of science courses. We focus specifically on a problem-solving physics task requiring mathematical curriculum from the service courses. This sort of challenging task requires complex higher-order thinking and utilization of problem-solving schemata.

The study presented here examines the processes involved in transfer, using a think-aloud method (Van Someren et al. 1994) to see how students articulate their thinking while attempting a task requiring transfer (task details are provided later). Think-aloud is a method that can be used to explore and examine the cognitive processes of thinking by asking participants to constantly verbalise what they are thinking while doing assigned tasks (Van Someren et al. 1994). Think-aloud reports provide rich information on cognition during complex problem solving tasks (Lohman 2000). This method was originally used in psychology (ibid.), and is currently applied in education, psychology, and learning science research on metacognitive skills (Bannert and Mengelkamp 2008), collaborative problem solving (Siddiq and Scherer 2017), the process of schematic representation (Anwar et al. 2018), web-based learning (Young 2005), and science text and diagrams (Cromley et al. 2010). However, in relation to mathematics education, there are few studies using think-aloud (e.g., Brennan et al. 2010; Ke 2008; Monaghan 2005) and these papers focus on primary school mathematics. We were unable to find prior research using the think-aloud method in the context of university mathematics education.

Analysis of the think-aloud accounts is framed by mathematical problem-solving theory (Mayer 1992; Okamoto 2008; Seo 2010) and also Bloom’s taxonomy (Bloom et al. 1956). We also use qualitative data from interviews to further explore student and academic perspectives on problem solving, transfer, and the broader relationship between mathematics and science learning. We address the following research questions:

1. What are the processes of transfer (if any) evident in students’ “think-aloud” accounts while solving physics exam questions requiring knowledge and understanding from their mathematics service courses?
2. What are the challenges in transfer of learning reported by students and by academics?
3. What teaching and learning factors do students and academics believe to enhance transfer?

## 2. Literature Review

We present a brief review of literature relating to transfer of learning, including important theoretical explanations of how transfer might occur and what factors are involved in it. The review focuses specifically on the research examining transfer in mathematical and science contexts, although a vast corpus of literature exists on transfer more generally.

### 2.1. Transfer of Learning

Many researchers in education and psychology have investigated transfer since the late nineteenth century. In a broad sense, as learning involves the application of prior learning to new similar or different contexts, transfer is related to all learning. Accordingly, transfer can be seen as the central or primary goal in education (Bransford et al. 1999; De Corte 1995, 2003; Engle 2012; Mestre 2003; Siler and Willow 2014). The acknowledgment that 21C learning will require adaption to rapidly evolving environments means that transfer of learning is key to efficient and effective education in the future. Teaching and learning that is able to optimise transfer of learning is an important goal. In their account of 21C learning, Saavedra and Opfer point out:

Students must apply the skills and knowledge they gain in one discipline to another and what they learn in school to other areas of their lives. A common theme is that ordinary instruction doesn’t prepare learners well to transfer what they learn, but explicit attention to the challenges of transfer can cultivate it.

(Saavedra and Opfer 2012, p. 10)

As transfer can be related to all learning, the list of factors associated with it can be extensive and we review here only some indicative starting points. Billing (2007) identifies nine major factors to facilitate transfer based on his survey of several hundred papers. They are (i) motivation, (ii) metacognitive strategies and skills, (iii) learning in context, (iv) principles, rules, and schemata acquisition, (v) similarity and analogy, (vi) varied examples and contexts, (vii) reduced cognitive load, (viii) active learning, and (ix) learning by discovery. In addition, transfer can be promoted by the necessity of prior knowledge (see Mestre 2003) and learning with deep understanding (Barnett and Ceci 2002; Brown and Kane 1988; Chi et al. 1989, 1994).

### 2.2. Cognitive Explanations of Transfer

Undoubtedly, transfer involves higher order thinking (Anderson et al. 2001; Bloom et al. 1956), as does mathematical problem solving (Mayer and Wittrock 1996). Consequently, to analyse transfer in the problem-solving task in this study, we use both a framework for mathematical problem solving (Seo 2010) and Bloom’s seminal cognitive taxonomy which outlines lower and higher-order thinking (Bloom et al. 1956).

#### 2.2.1. Higher Order Thinking and Bloom’s Taxonomy

The taxonomy of educational objectives developed by Bloom and colleagues (Anderson et al. 2001; Bloom et al. 1956; Krathwohl et al. 1964) has been influential in providing the foundations to understand transfer of learning (Krathwohl 2002). Bloom’s taxonomy (1956), which is one of the most seminal books in education (Anderson 2002), presents a framework that is used for various purposes, including assessment, curriculum development, instruction, and learning theory (Seaman 2011). Importantly, Bloom’s taxonomy is a useful tool to understand the different levels of cognitive processes related to higher order thinking. The taxonomy recognises three major domains in cognitive functioning: the cognitive domain (Bloom et al. 1956), affective domain (Krathwohl et al. 1964), and psychomotor domain. The first domain is relevant to this study as it is helpful to classify lower and higher order thinking. The cognitive domain entails “the recall or recognition of knowledge and the development of intellectual abilities and skills” (Krathwohl 2002, p. 7). The taxonomy categorises and ranks increasing levels of higher order thinking: (i) knowledge, (ii) comprehension, (iii) application, (iv) analysis, (v) synthesis, and (xi) evaluation. The taxonomy hierarchy is cumulative and easy to understand, with many examples listed by Bloom and colleagues (1956), a wealth of supporting empirical studies (e.g., Kropp et al. 1966; Miller et al. 1979), and well established validity (Seaman 2011). A summary, with explanations and some mathematical examples, is shown in Table 1. Research suggests that learners need to use all these cognitive processes, including the higher order thinking, for effective transfer of learning.

In mathematics education, Bloom’s taxonomy has been widely used for teaching and assessment for over a half century (Thompson 2008). However, it has faced criticism with claims that the taxonomy fails to distinguish between the different levels of mathematical reasoning (Suzuki 1997). Another issue is its apparent inaccuracy in predicting which of the cognitive processes students use to solve problems in mathematics tests (Gierl 1997). To address these weaknesses when analysing transfer of mathematical learning, we use Bloom’s cognitive taxonomy alongside more specific theory of mathematical problem-solving.

#### 2.2.2. Mathematical Problem-Solving Theories and Transfer

We use mathematical problem-solving theory to analyse students’ accounts of the transfer task to address the criticisms of Bloom’s taxonomy, and also because the nature of the exam questions used in the task is built around a problem-solving approach. 

Problem-solving is defined as “cognitive processing directed at achieving a goal when no solution method is obvious to the problem solver” (Mayer and Wittrock 1996, p. 47). Sub-processes of problem-solving cover representation, planning, and executing (Mayer and Wittrock 1996), all of which can involve metacognition which is “cognition on cognition” or “thinking about thinking.” Metacognition enhances student performance via conscious and deliberative problem-solving strategies in planning, monitoring, and evaluating (Ali et al. 2018). In order for successful transfer to occur, a learner selects appropriate previous skills and knowledge, applies them into new problems, and monitors the appropriate general and specific cognitive processes to solve the problems (Mayer and Wittrock 1996).

**Figure 1:**
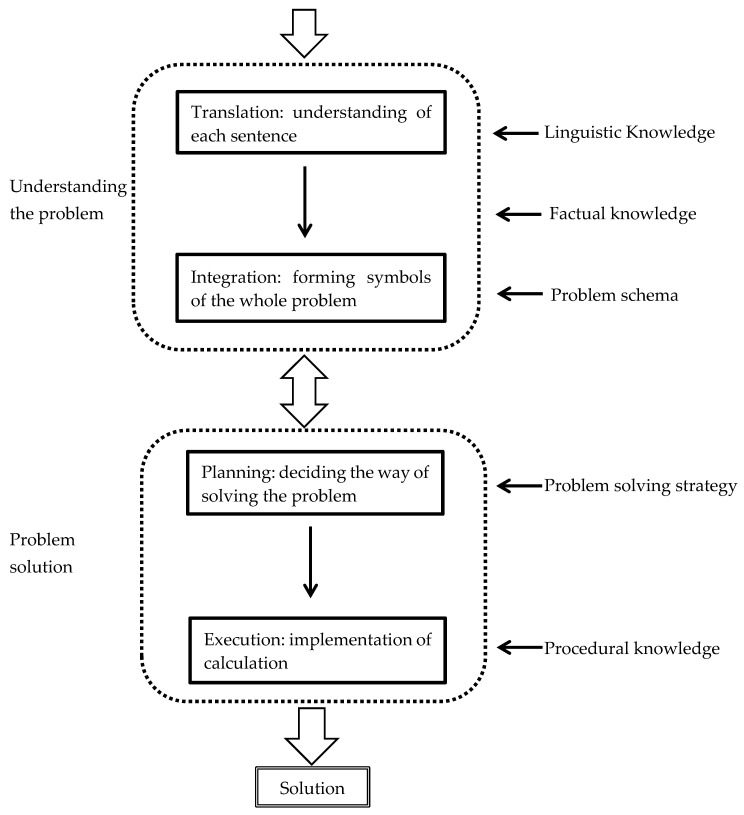
Four levels of procedures of mathematical problem solving. (Source: Seo 2010, p. 230 translated by Yoshitaka Nakakoji).

Literature on mathematical problem-solving falls into three categories (Okamoto 2008): mere calculation problems (e.g., Brown and Burton 1978), algebraic word problems (e.g., Kintsch 1986), and geometric problems (e.g., Owen and Sweller 1985). Metacognition is thought to be more deeply involved with word problems and geometric problems than calculation problems (Okamoto 2008). Some literature provides additional detail on four levels of cognitive processes in this mathematical problem solving (e.g., Mayer 1992; Okamoto 2008) and a specific model of cognitive sequencing and flow, see Figure 1, which is translated from Seo (2010).

In this study, we use Seo’s model as a theoretical framework, alongside Bloom’s taxonomy, to analyse the processes of transfer. Whilst Seo’s model comes from the Japanese literature on mathematical learning, it shows some remarkable similarities to the OECD PISA problem solving assessment framework (DeBortoli and Macaskill 2014). The model covers basic procedural steps, and it was anticipated that this could frame students’ descriptions in their think-aloud accounts. This was considered appropriate for a first pass application of think-aloud to transfer, some of the more complex models of problem solving (e.g., Ichikawa et al. 2009) may be appropriate for follow-up research.

In particular, a problem schema is seen as important in mathematical learning (Okamoto 2008; Silver 1987) and is defined as patterned knowledge about structures of problems and ways of solving problems (Seo 2010). The schema referred to in Seo’s model is “a cluster of knowledge representing a particular generic procedure, object, percept, event, sequence of events, or social situation” (Thorndyke 1984, p. 167), which is featured by five characteristics: abstraction, instantiation, prediction, induction, and hierarchical organization (Reed 1993). This schema is understood to be especially useful in solving word problems (Seo 2010) and geometric problems (Okamoto 2008). Students with insufficient problem schema may experience mathematical learning difficulties. Many primary and secondary students, although good at calculations, have difficulties with word problems (Seo 2010). This is because they fail to understand the meaning of problems and form the representation of the whole problems (Seo 2010). This is related to the translation and integration procedures in Figure 1, which involve use of a problem schema. According to schema theory, transfer of learning is heavily subject to whether appropriate anticipatory schemata are activated (Salomon and Perkins 1989). For example, vertical transfer (transfer of basic knowledge to higher level understanding) needs to activate procedural schemata which have been developed previously (Royer 1979).

### 2.3. Socio-Cultural Explanations of Transfer

Socio-cultural theories of transfer emphasise the importance of social and cultural learning interactions and contexts. In particular, situated learning (for example, see Greeno 2011; Greeno et al. 1993; Lave 1988; Lave and Wenger 1991) and the actor-oriented approach to transfer (Karakok 2009; Lobato 2006, 2008a, 2008b) are relevant to transfer of mathematical learning. In this study, these perspectives are useful for understanding the context of transfer in mathematics and science education at university. These theories highlight the importance of individuals’ personally constructed learning and the interplay between their understanding of mathematics and the process of solving transfer tasks.

Lave (1988) first pointed out a paucity of transfer studies in natural settings and academic disregard of the need for problem-solving in daily life situations. Lave’s research on situated learning makes it clear that learners, their thinking, and learning activities are not independent from their contexts, thus “cognition and performance are context-specific, in a fundamental sense” (Evans 1998, p. 270). Greeno et al. (1993) expanded and articulated the situatived perspective on transfer, emphasising not only the importance of the situation where learning occurs, but also the learner’s ability to interact with other people and the various materials available for learning (Marton 2006). In relation to our study, this perspective acknowledges that while mathematical learning occurs in formal course settings, including university lectures and tutorials with interactions between lecturers, tutors, and peers, but we cannot view this learning as complete. In addition, learning can be constructed when students study by themselves, or with others, at home, in a library, or elsewhere, and by utilising physical and online materials.

In experimental studies, researchers have highlighted difficulties in observing transfer (see for example, Detterman 1993; Hatano and Greeno 1999; Lobato 2006). The sociocultural actor-oriented approach attempts to overcome this difficulty, and some other weaknesses in understanding transfer, by shifting to a learner-centred perspective. This approach specifically examines transfer processes by looking at how learners relate to learning experiences within novel situations. Thus, this approach enables researchers to consider the occurrence of transfer even if students provide incorrect or non-standard performance in tasks, while this situation would be treated as failure of transfer in experimental studies where transfer is measured as a dichotomised absolute. Adopting an actor-oriented approach, Karakok (2009) identified transfer of students’ understanding of the concepts in linear algebra to quantum physics contexts. In this paper, although we utilise cognitive theoretical frameworks for analysis of individuals’ account of transfer processes, socio-cultural perspectives, acknowledging individuals own constructions of their learning, and the importance of context, frame the study more fully.

## 3. Methods 

### 3.1. Research Design

There are two data collection strategies employed. First, student interviews look at both the processes of transfer tasks, using a think-aloud method, and a post-task interview data is also gathered on students’ perceptions about the relationship between mathematics and science and the issues related to transfer. Second, interviews with academic teaching staff explored the relationship between mathematics and science; and factors promoting and hindering transfer.

### 3.2. Student Think-Aloud Study

We examined first-year university students’ transfer processes by giving them physics exam questions and getting them to think-aloud while they completed this task. Post-task interviews were conducted with questions exploring student perspectives of mathematics and science learning and transfer between these.

#### 3.2.1. Sample

Ten students in STEM degrees at an Australian university agreed to participate in this study. We purposively selected student cohorts studying first semester mathematics service courses and second semester physics courses and recruited volunteers from classes. Relevant background information was collected: age (mode = 21, range 19–32), gender (female 30%), and degree (all were Bachelor of Science, three in advanced courses and two in degrees combined with arts or education).

#### 3.2.2. Data Collection

Individual interviews were conducted to collect the following information. First, a short half-page questionnaire was used to collect the background information of students. Second, a cognitive interview was conducted using the think-aloud method to examine the learning processes of transfer; this is explained in detail in the following sections. A third element was a post-task interview used to identify the strategies used and difficulties faced by students in the transfer task. 

#### 3.2.3. Think-Aloud Tasks

**Figure 2:**
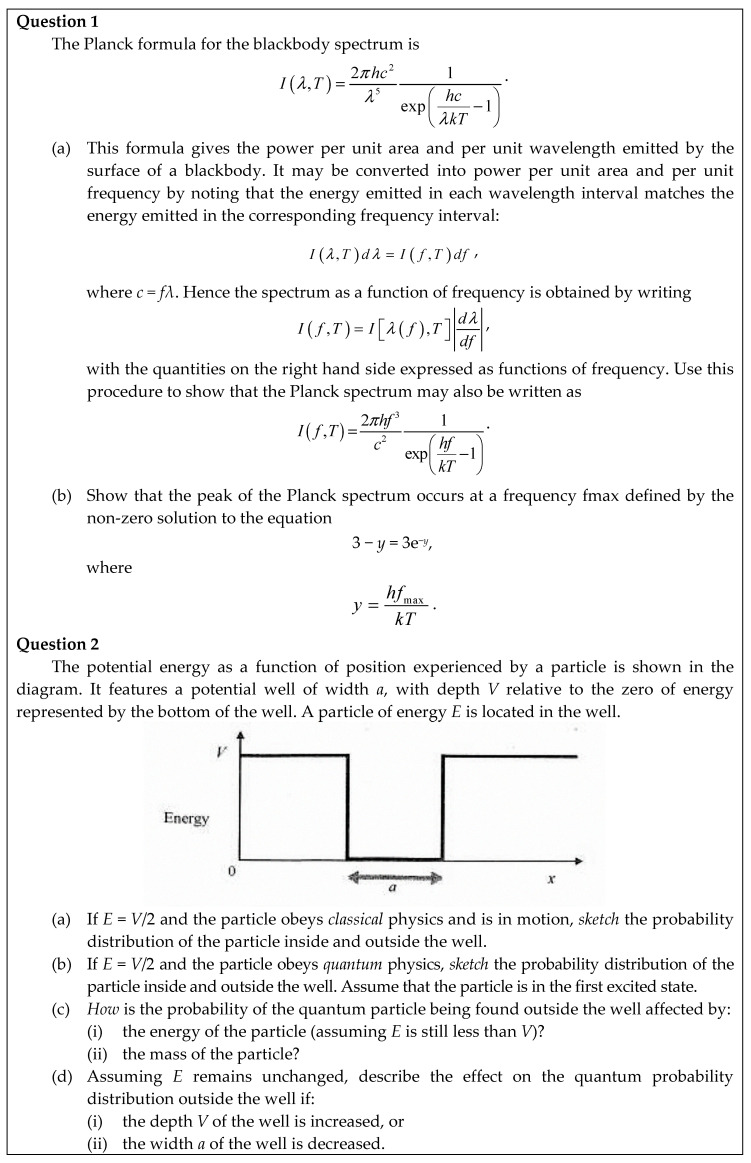
Think-aloud physics Questions 1 and 2 used for examination of transfer processes.

Students were asked to speak aloud about what they were thinking, while solving two physics questions extracted from first year second semester past exams (see Figure 2). These physics questions required mathematical skill, knowledge, and reasoning learned in mathematics service courses. The first question involved calculation of a partial derivative. The second question could be mathematically answered by solving Schrödinger equation; however, the application of mathematics to solve the second question was not obvious and an intuitive approach to physics could be employed as an alternative. To extract more insight into the students’ thinking, a follow-up interview was also used to retrospectively explore how the question was attempted.

#### 3.2.4. Data Analysis

In order to look at the processes of transfer, the six categories in Bloom’s taxonomy (see Table 1) and the four levels of processes of mathematical problem-solving (based on Seo 2010, see Figure 1) were used as descriptive categories for the various processes students described in their think-aloud accounts. Drawing on both the think-aloud transcripts and the working out evident on the transfer task answer sheets, each process was also coded by the language used (words and phrases), mathematical expressions, formulae, or graphs (see some examples in Table 2). In addition, qualitative thematic analysis was used to identify themes generated from the post-task interview data.

### 3.3. Interviews with Academics

An interview survey was conducted to ascertain academic practitioners’ views on teaching and learning. The academics were experts in their own research fields, and experienced in higher education teaching and learning. Almost all of them held post-graduate qualifications in higher education teaching and learning and they were able to provide informed and articulate comment on learning issues and challenges. However, it needs to be acknowledged and emphasized that whilst they provided a range of authentic practitioner perspectives, none were expert on transfer of learning. Academic teaching staff had been involved in, and interviewed, previous teaching and learning research, but not research on transfer of mathematical learning. This study addresses that gap with a preliminary, small sample.

#### 3.3.1. Sample

The sample included eight senior teaching academics across four disciplines (5 mathematics, 1 IT, 1 physics, and 1 bioscience; 4 males and 4 females) who were invited to participate in this study and were required to meet the three conditions: (i) knowledge and experience with the issues under investigation; (ii) capacity and willingness to participate; (iii) sufficient time to participate in the interviews.

#### 3.3.2. Data Collection and Analysis Methods

There were two rounds of interviews asking experts fifteen open-ended questions in total. In the first round, sample questions covered “What mathematical knowledge and skills taught in first year mathematics do you think are most relevant to study in biology, biochemistry, engineering and physics?” and “What factors enhance or hinder students’ application of mathematical skills and knowledge in biology, biochemistry, engineering and physics?” On the basis of analysis of responses, a second round of questions were made for clarification. For their convenience, participants were invited to answer these questions by e-mail. This email interview technique has been used in other studies on teaching and learning mathematics (Petocz et al. 2006). The interview data transcribed was analysed with using thematic analysis, according to the principles outlined by Braun and Clarke (2006).

## 4. Results and Discussion

We present our findings and discussion around the four research questions.

### 4.1. Research Question 1: What Are the Processes of Transfer (If Any) Evident in Students “Think-Aloud” Accounts While Solving Physics Exam Questions Requiring Knowledge and Understanding from Their Mathematics Service Courses?

**Figure 3:**
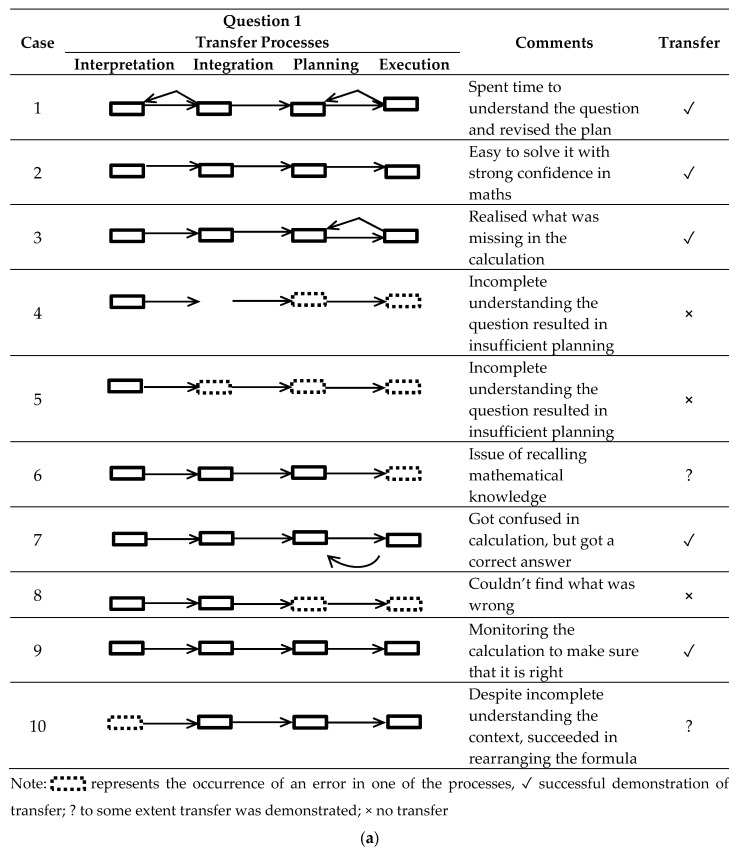

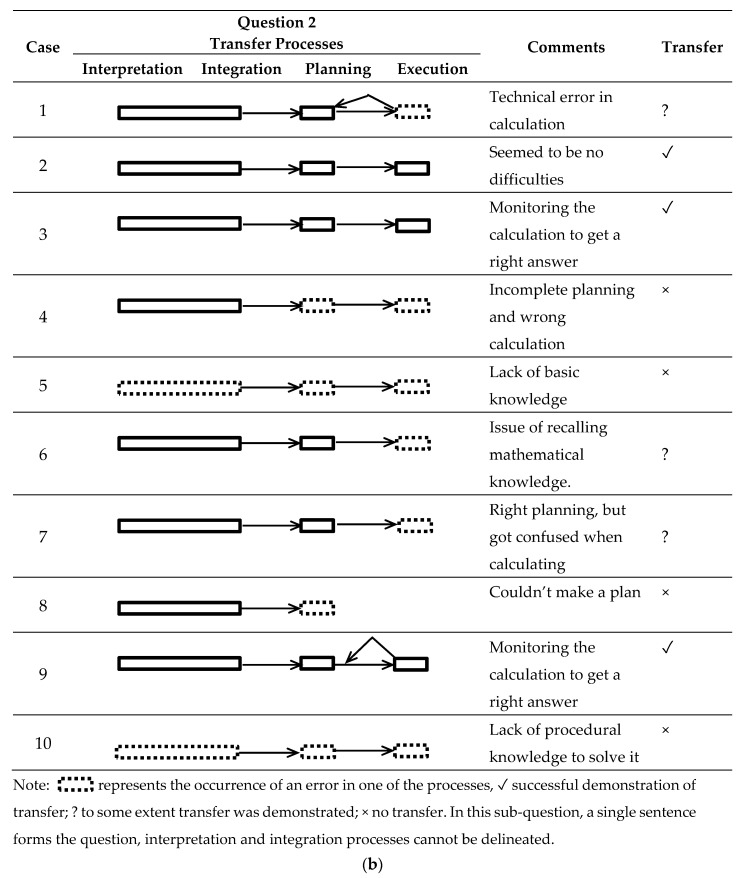
(**a**) Think-aloud reports on transfer processes for question 1. (**b**) Think-aloud reports on transfer processes for question 2.

For each student, their progression through the elements of Seo’s theory (2010) interpretation, integration, planning, and execution was coded and presented in a temporal model (see Figure 3a for question 1(a) and in Figure 3b for question 1(b). The coding of the processes was based on categorisation of phrases and synonyms relating to each of Seo’s four processes. For example, the students’ think-aloud account was categorised as *interpretation* if they reported “I’m reading the question and I’m thinking about the relationship between frequency and wavelength” (Student 1) or planning if they stated “we just need to replace in the formula where the wavelength is used, … and … to multiply it by dλ df” (Student 2).

To interpret Figure 3a,b, the reader can view each students’ progression, and/or recursive movement, from left to right; this reflects their reported thinking over time as they attempted to answer the physics question. Comments provide additional important information and the transfer column highlights whether any transfer of mathematics learning to the physics task was evident. All students followed the interpretation, integration, planning, and execution processes highlighted in Seo’s model. But, there was one small exception, student 4, who missed the integration process. Further, some students (e.g., 5) made errors at various points (see dotted line boxes) and others still (e.g., 1) took recursive steps to modify their approach to the problem when they made initial errors.

The extent to which transfer was demonstrated varied among students and this may be related to question difficulty. In the sub-question 1(a), five students out of ten got correct answers, therefore demonstrating transfer, while in the sub-question 1(b) only three students managed this. The small proportion demonstrating transfer in the latter question was not surprising given the increased complexity of the calculation. Two students in the former and three students in the latter also were able to demonstrate understanding related to transfer to some extent; socio-cultural theories of transfer suggest this can be considered partial transfer.

This variation in how transfer was demonstrated among students is consistent with socio-cultural studies on transfer, in particular the actor-oriented approach to transfer (see for example, Karakok 2009), which highlights diversity in how transfer of learning is constructed within individuals learning. Furthermore, analysis of the think-aloud accounts made it evident that metacognition was also an important part in all four of the transfer processes. For example, students reviewed their planning and calculation, some of them more than once, and this reflects their conscious monitoring of their own thinking.

There were students who couldn’t solve the questions. For example, some students had the right approach, but couldn’t fully demonstrate transfer (see cases 6 and 7 in Figure 3b). It is important to consider what made the questions difficult for these students. There were five main issues identified from the analysis of the transfer processes: (i) lack of mathematical knowledge, related to the first and foundational category of Bloom’s taxonomy—knowledge; (ii) difficulties in understanding the question, i.e., issues in translation and integration processes, related to the second category of Bloom’s Taxonomy—comprehension (iii) issues in recalling prior learning, which are also related to the first category of Bloom’s taxonomy as well as planning and execution processes; (iv) a lack of procedural knowledge to solve the question—or poor use of problem schema; (v) a lack of practice and/or a technical error in calculation, related to the execution process. In the post-task interviews, students mentioned difficulties in understanding the problem, for example:

“in the first question with the Planck’s formula, it’s difficult to understand the question to begin with. So reading through the question is a lot to figure out. And you also have to recognise lots of different symbols, maths symbols which I’m sure if I didn’t know what they were I would be very lost, even more than I was.”

In regard to problem schema, another student also stated that “I understand what it’s asking me to do. I just don’t know how to do it.” These issues, mentioned by students, are consistent with the difficulties in mathematical learning outlined by Seo (2010). These difficulties are orientated to the nature of the question task as questions 1(a) and (b) relied mostly on calculation. However, as we will explain, these contrasted substantially with difficulties in Question 2, which had much greater demands on reasoning abilities.

Question 2 (see Figure 2), required thinking about probability distributions and exponential decay, however, no students were able to solve this question using mathematical formula in physics or mathematical reasoning in an explicit way. This led to difficulty in conducting the analysis and transfer could not be observed. For question 2, the students preferred recalling their basic knowledge of physics and tended to utilise an intuitive approach to solve the physics questio—rather than employing the mathematical methods taught in their service course in the previous semester. Although it is important for students to understand the concepts in physics in their disciplinary ways, the understanding of mathematical expressions behind the physical world is equally important and of perhaps greater utility in terms of development of their generic 21C skills.

The students’ inability to approach Question 2 with mathematical understanding may be related to a disparity between mathematical service courses and physics courses, wherein the content taught in mathematics classes is assumed and not reinforced within the physics classes. In other words, conceptual understanding in physics may be overemphasised or presented without in-depth exploration and reinforcement of the relevant mathematical aspects. This issue was touched on by one academic who suggested in interview: “I suppose there is so much to test conceptually in the sciences in an examination that educators do not want students to spend time on mathematical working.”

It is impossible to verify if this was the case. We can only report that for Question 2, the analysis of transfer of mathematical learning was not possible because mathematical reasoning was not evident in the students’ think-aloud accounts.

### 4.2. Research Question 2: What Are the Challenges in Transfer of Learning Reported by Students and by Academics?

Interviews explored students’ and academics’ perspectives on what would hinder and enhance transfer of learning. This was done through a series of open-ended questions which generated a large amount of data. After coding students and academics responses separately and producing highly synthesised themes, we compare and contrast these in Table 3.

The factors reported here were consistent with a range of educational research studies and theories. For example, Hattie’s (2009) syntheses of meta-analyses in education also highlights teacher clarity and feedback as among the most influential teaching factors. Unsurprisingly, mathematical anxiety was mentioned by both students and academics. Academics are aware of their students’ mathematics anxiety: “I imagine this is to do with a perceived lack of mathematical understanding, and fear of mathematics, among students (and society in general)” (Academic 2). Both students’ and academics’ comments on mathematics anxiety made it clear that this was an issue central to teaching and learning. We wondered if students’ fear of mathematics and academics’ sensitivity to this fear was related to the low level of science academics’ inclusion of mathematics in science assessments. We reviewed a range of science exam papers in the university and found there were no questions aligning with the tertiary mathematics taught in mathematics service courses in either biology or bio-chemistry, despite the fact that students were required to undertake those university mathematics courses. In physics, just a few questions were aligned with the university mathematics service courses, with high-school level mathematics (advanced and extension courses) more evident in the first-year physics papers. If mathematical learning is not assessed in the science disciplinary context, why are mathematics courses compulsory for science students at university? Unfortunately, in the interviews academics provided no direct replies to this question. What was evident is that first, both students and academics acknowledge the importance of mathematics, but academics think that understanding concepts in their disciplines is more important than mathematical application. Second, scientist academics think that mathematical skills and understanding should be assessed in mathematics service courses, not science courses. Academics also acknowledged that it is very problematic and difficult to provide mathematics courses to accommodate learning needs for students from diverse backgrounds and disciplines, such as science and engineering.

Finally, “translation” emerged as an important theme for academics (see Table 3), and although students did not mention it in interview, it did emerge as an issue for students in the think-aloud task. Translation was seen as important as science problems with mathematical content can be expressed in the form of word problems. One academic commented: “the most difficult problem for many students is not with the maths but converting from words to maths and back” (Academic 3). Our analysis of the transfer processes showed translation was an important strategy for successful transfer of learning and problem-solving (see student 1 in Figure 3a).

### 4.3. Research Question 3: What Teaching and Learning Factors Do Students and Academics Believe to Enhance Transfer?

Interviews also explored students and academic perspectives on what would enhance transfer of learning, shown in Table 3. Overall, the reported factors are consistent with a range of educational theories; for example, transfer theory suggests that higher order thinking, like application of understanding in real world problems and rehearsal (repeated practice) are enablers of transfer. Furthermore, students’ self-beliefs and confidence are important in learning in general (Malmberg et al. 2013); as is prior learning (Martin et al. 2013). Thus early experiences in mathematical learning are likely to be a key to successful transfer of mathematical understanding in university.

There was one noticeable gap between student and academic perspectives. While academics expect students to: “try to see wider connections and the historical development of mathematics in science, rather than only focus on a narrow disciplinary context” (Academic 4); the student perspective suggests that in relation to science learning “the basics of maths that I’ve done [are] very useful, but the stuff I’m doing right now is not quite so” (Student 10). In interviews, academics identified the relevance of mathematics and interdisciplinary learning as important; by contrast, students did not mention these as important to promoting transfer (Table 3). This demonstrates an apparent gap in how the utility of mathematics is perceived by students and academics. Socio-cultural theories of transfer suggest that students need to value learning in order to transfer it effectively. If academics are not able to communicate the value and potential of the mathematics learning, this presents a barrier to transfer.

There are factors identified in research as important to transfer, which were not apparent in these academics’ and students’ responses. First, although socio-cultural theories emphasise the importance of learning contexts, none of the interview respondents mentioned contextual factors like interaction with lecturers, tutors and/or peers in lectures, tutorials, or other situations and material available for learning.

Also unexpectedly, neither students nor academics mentioned metacognition in the interviews. This is despite the fact that research literature views metacognitive knowledge and activities, such as monitoring and control, as essential to mathematical learning and transfer (Billing 2007; Mayer and Wittrock 1996; Okamoto 2008; Seo 2010). It was evident in think-aloud that students employed metacognitive strategies; for example, when monitoring calculation a student stated “Did I do something wrong?” (Student 3). However, they may not have been aware of this strategy and did not discuss this in the post-task interviews. As education systems shift to focus on transferability of generic skills, they are likely to promote a stronger focus on metacognitive skills.

## 5. Conclusions

Although it is ubiquitous to all conceptions of learning, “transfer of learning” is in keener focus within the modern conception of 21C skills due to their aims to provide generic skills and competencies. Such skills need to be carried and applied, or transferred, to a wide range of contexts. It is anticipated that the demands of future learning, within rapidly changing environments, will require increasing competence in transfer of learning. Our experience exploring “transfer of learning” between mathematics and science at one university has highlighted a range of issues and possibilities.

Firstly, through this study, we were able to demonstrate how a process-oriented approach (Sternberg 2000) to learning could be applied in an authentic educational context, documenting student thinking during a mathematical problem-solving transfer task. Although small in scale, the findings demonstrate how a larger study using this approach could be used to provide diagnostic information to strengthen teaching and learning. Using Seo’s problem-solving theory and Bloom’s taxonomy, students’ thinking pathways and stumbling blocks in this process could be analysed by teacher academics. Common difficulties can be identified and teaching and learning designed to rectify them. While diagnostic assessments for school and adult literacy and numeracy have been available for decades, there remains potential to develop similar methods for practitioners teaching for 21C skills, including transfer of learning. There are already a range of assessments of problem-solving skills that might be adapted to this purpose.

Our findings provide the opportunity to reflect on models for the teaching of mathematics in universities. We argue that transfer of learning between mathematics and science is a neglected area of enormous potential. Given societal demands for increasing STEM capacity, it is critical and inevitable that we reflect on the following questions: What are the key challenges in mathematics learning for sciences? How should mathematics be taught to university science students? Transfer of learning, and associated cognitive and socio-cultural theories, provide a conceptual framework through which these questions can be considered and explored.

The close relationship between mathematics and intellgience factors, the demands for numeracy and data skills from industry, and the potential of mathematics in problem-solving all suggest that reforms in mathematics education are needed and should be discssed as part of the 21C skills agenda. There are currently different models for higher education mathematics across countries and institutions. For some countries, such as Australia, mathematics service courses are provided, particularly for first year undergraduate students. These aim to enable students to apply mathematics to diverse contexts, but might make it difficult for students to see the connection with their own discipline. In other countries, such as Japan, it is more common that each discipline teaches mathematics, and courses are embedded into each discipline’s degree programs. This may help students to apply mathematics to their own disciplinary context, but may restrict the applicability of mathematics to more diverse contexts. Investigations into the effectiveness of each approach would be useful, as would broader contemplation of how mathematics might be positioned within interdisciplinary learning and strategy for developing 21C skills.

Finally, this project has led us to believe that greater attention could, and should, be paid to the concept of “transfer of learning” in order to promote 21C skills. While there is a long literature on transfer, there are enormous gaps in relation to how transfer might be measured, evaluated, and promoted within schools and universities. Now is the time to bring what we know of transfer into the realm of educational practice; and where understanding is lacking we must develop a program of research. In particular, interdisciplinary learning, which has been positioned as a key goal for the future (as technology and industry developments are expected to morph and transform traditional disciplinary boundaries) has not been thoroughly explored at the level of institutional practice.

While 21C skills lauded as critical for the new century include problem-solving skills, and recent educational rhetoric continues to exhort the value of integrated interdisciplinary learning, particularly in STEM, there remains a need for research examining if, and how, pursuit of these goals is evident in current educational practice.

## Figures and Tables

**Table 1:** Summary of Bloom’s original taxonomy (1956) with mathematics application examples.

Classification	Explanation	Examples
1. Knowledge (i) Knowledge of specifics (ii) Knowledge of ways and means of dealing with specifics (iii) Knowledge of the universals and abstractions in a field	Knowledge contains behaviours and tests situations which emphasize the remembering, either by recognition or recall, of ideas, material, or phenomena (p. 62).	(i) To define technical terms in mathematics by giving their attributes, properties, or relations. (ii) Knowledge of the mathematical techniques and methods used by scientists in seeking to answer questions about the world. (iii) The recall of major theorems in mathematics (pp. 64–77).
2. Comprehension (i) Translation (ii) Interpretation (iii)Extrapolation	The emphasis is on the grasp of the meaning and intent of the material (p. 144).	(i) The ability to translate abstract concepts in mathematics by giving an illustration or example. (ii) The ability to interpret various types of numerical data. (iii) Skill in predicting continuation of trends using mathematical models. (pp. 92–96).
3. Application	Remembering and bringing to bear upon given material the appropriate generalizations or principles (p. 144)	(i) The ability to apply scientific principles, postulates, theorems in mathematics, or other abstractions to new situations (ii) The ability to apply the laws of trigonometry to practical situations
4. Analysis (i) Analysis of elements (ii) Analysis of relationships (iii) Analysis of organizational principles	Emphasizes the breakdown of the material into its constituent parts and detection of the relationships of the parts and of the way they are organized. (p. 144)	(i) Ability to distinguish a conclusion from statements which support it. (ii) Ability to detect logical fallacies in arguments and proof in mathematics. (iii) Ability to recognize form and pattern in mathematics as a means of understanding their meaning (pp. 146–48)
5. Synthesis (i) Production of a unique communication (ii) Production of a plan, or proposed set of operations (iii) Derivation of a set of abstract relations	Definition: the putting together of elements and parts so as to form a whole. In comparison with the subordinate classifications, this classification is less practical and more emphasis on uniqueness and originality. (p. 162)	(i) Skill in writing, using an excellent organization of ideas and statements in mathematics. (ii) Ability to integrate the results of an investigation into an effective plan or solution to solve a problem in mathematics. (iii) Ability to make mathematical discoveries and generalizations. (pp. 169–72)
6. Evaluation (i) Judgments in terms of internal evidence (ii) Judgments in terms of external evidence	Definition: the making of judgments about the value, for some purpose, of ideas, works, solutions, methods, material, etc. In addition, the process may require behavior classified in other subordinate categories (p. 185)	(i) Judging internal standards, the ability to assess the quality of quantitative data analysis in relation to the experiments conducted. (ii) The comparison of major theories, generalizations and facts in mathematics and science. (Based on pp. 189, 192)

Source: (Bloom et al. 1956).

**Table 2:** Examples of words, phrases, and mathematical expression associated with coding four processes in solving transfer questions 1 and 2.

Processes	Words or Phrases	Mathematical Expression
1. Interpretation	I know the meaning of I’m not sure about	*c* = *f*λ *y* = exp (−*kx*), where *k* > 0
2. Integration	This question means I can’t understand the question.	Requires rearrangement of *I* (*f*, *T*) Understanding of a graph of *y* = exp (−*kx*), *k* = (√2*m* (*V*−*E*))/ℏ
3. Planning	I know how to do Show No idea about	*∂I*/*∂f* = 0 at *f* = *f*_max_ Preparation of drawing the graph with considering how it changes with respect to *m*, *V*, and *E*.
4. Execution	Substitution of into (Partial) derivative of	λ = *c*/*f*, dλ/d*f* = −*c*/*f*^2^ Actual sketch of the graph

**Table 3:** Potential factors promoting or hindering transfer.

	Factors Enhance Transfer	Factors Hinder Transfer
Academics’ perspective	Interdisciplinary learning Showing relevance of maths Practice Confidence & self-belief Prior leaning	Anxiety Poor feedback Poor explanation Translation Disparity of pedagogy Mismatch of expectation Surface learning approaches Poor maths preparedness
Students’ perspective	Importance of understanding the question Memory or recall Prior learning Intuition	Anxiety Mismatch of expectation Difficulty in understanding the problem Difficulty in recalling maths knowledge & skills
